# Di-μ-acetato-κ^3^ *O*,*O*′:*O*′;κ^3^ *O*:*O*,*O*′-bis­[(acetato-κ^2^ *O*,*O*′)bis­(5-nitro-1,10-phenanthroline-κ^2^ *N*,*N*′)cadmium]

**DOI:** 10.1107/S1600536811032259

**Published:** 2011-08-17

**Authors:** Fenghua Cui, Shuxia Zhang

**Affiliations:** a Department of Chemistry, Henan Normal University, Xinxiang 453007, People’s Republic of China; b Department of Engineering and Technology, Xinxiang Vocational and Technical College, Xinxiang 453007, People’s Republic of China

## Abstract

In the binuclear title compound, [Cd_2_(C_2_H_3_O_2_)_4_(C_12_H_7_N_3_O_2_)_2_], the Cd^II^ cations are linked by carboxyl­ate O atoms into a four-membered Cd_2_O_2_ rhombic ring with a Cd⋯ Cd separation of 3.7515 (5) Å. Each Cd^II^ atom is seven-coordinated by a bidentate 5-nitro-1,10-phenanthroline (5-NO_2_-phen) ligand and two bidentate acetate anions, one of which also acts as a bridge linking the two Cd atoms. The crystal packing is stabilized by π–π inter­actions between the phen rings of neighboring mol­ecules, with centroid–centroid distances of 3.491 (2) (intra­molecular) and 3.598 (2) Å (inter­molecular).

## Related literature

For related structures, see: Peng *et al.* (2008 ▶); Harvey *et al.* (2008 ▶); Kruszynski *et al.* (2009 ▶). For our studies on transition metal complexes with 1,10-phenanthroline (phen) and its derivatives and carboxyl­ates, see: Xuan *et al.* (2007*a* ▶,*b* ▶, 2008 ▶). For their applications in organic transformations, mol­ecular recognition and organization of mol­ecular solids, see: Braga *et al.* (1998 ▶).
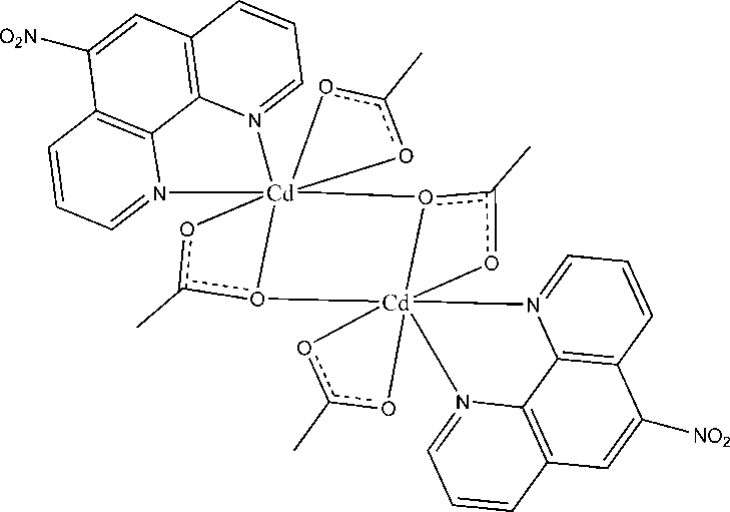


## Experimental

### 

#### Crystal data

[Cd_2_(C_2_H_3_O_2_)_4_(C_12_H_7_N_3_O_2_)_2_]
*M* *_r_* = 911.39
Monoclinic, 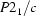

*a* = 18.653 (3) Å
*b* = 11.2236 (16) Å
*c* = 15.510 (2) Å
β = 94.531 (2)°
*V* = 3236.9 (8) Å^3^
*Z* = 4
Mo *K*α radiation
μ = 1.39 mm^−1^
*T* = 298 K
0.45 × 0.30 × 0.27 mm

#### Data collection

Bruker SMART CCD area-detector diffractometer
Absorption correction: multi-scan (*SADABS*; Bruker, 1997 ▶) *T*_min_ = 0.573, *T*_max_ = 0.705
17895 measured reflections
5988 independent reflections
4970 reflections with *I* > 2σ(*I*)
*R*_int_ = 0.022

#### Refinement

*R*[*F* ^2^ > 2σ(*F* ^2^)] = 0.025
*wR*(*F* ^2^) = 0.057
*S* = 1.02
5988 reflections
473 parameters
H-atom parameters constrained
Δρ_max_ = 0.53 e Å^−3^
Δρ_min_ = −0.36 e Å^−3^

### 

Data collection: *SMART* (Bruker, 1997 ▶); cell refinement: *SAINT* (Bruker, 1997 ▶); data reduction: *SAINT*; program(s) used to solve structure: *SHELXS97* (Sheldrick, 2008 ▶); program(s) used to refine structure: *SHELXL97* (Sheldrick, 2008 ▶); molecular graphics: *SHELXTL* (Sheldrick, 2008 ▶); software used to prepare material for publication: *publCIF* (Westrip, 2010 ▶).

## Supplementary Material

Crystal structure: contains datablock(s) I, global. DOI: 10.1107/S1600536811032259/bg2418sup1.cif

Structure factors: contains datablock(s) I. DOI: 10.1107/S1600536811032259/bg2418Isup2.hkl

Additional supplementary materials:  crystallographic information; 3D view; checkCIF report

## Figures and Tables

**Table 1:** Selected bond lengths (Å)

Cd1—O6	2.324 (2)
Cd1—N2	2.372 (2)
Cd1—O9	2.378 (2)
Cd1—O7	2.390 (2)
Cd1—N1	2.403 (2)
Cd1—O5	2.423 (2)
Cd1—O8	2.429 (2)
Cd2—O12	2.238 (2)
Cd2—O9	2.300 (2)
Cd2—O8	2.365 (2)
Cd2—N4	2.394 (2)
Cd2—N5	2.435 (2)
Cd2—O11	2.527 (3)
Cd2—O10	2.590 (3)

**Fig. 1.**
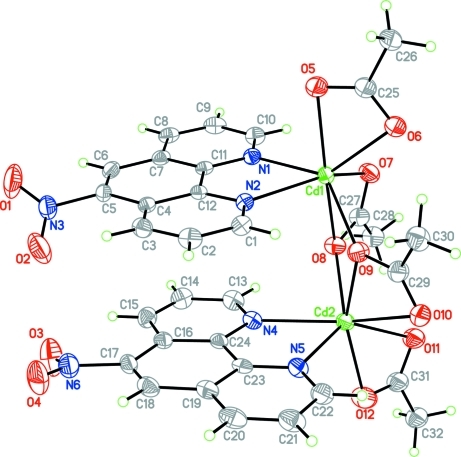
The molecular structure of the title compound, with atom labels and 30% probability displacement ellipsoids for non-H atoms.

**Fig. 2.**
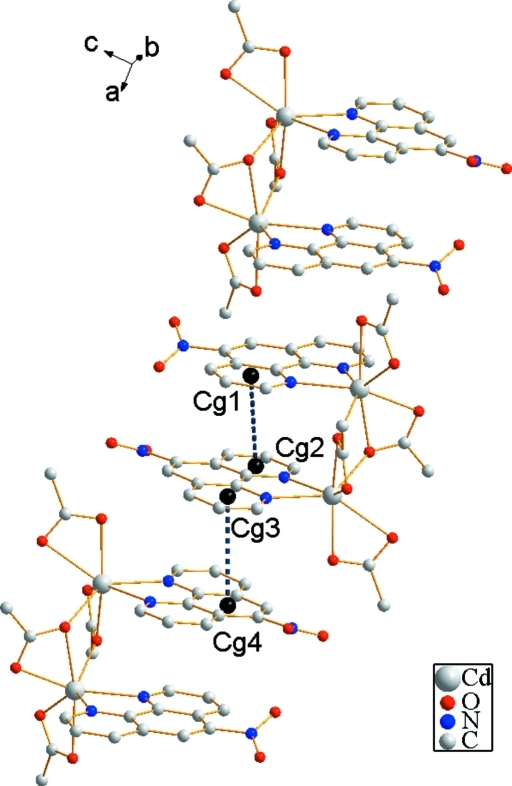
π-π interactions between the aromatic rings of the title compound. H atoms have been omitted for clarity.

## supplementary crystallographic information

### Comment

The work presented here is a continuation of our studies on transition metal complexes with 1,10-phenanthroline (phen) and its derivatives and carboxylates (Xuan *et al.* 2007*a*, 2007*b*, 2008), due to their applications, for example in organic transformations, molecular recognition and organization of molecular solids (Braga *et al.* 1998). Among such compounds, a number of dinuclear cadmium(II) compounds with 1,10-phenanthroline (phen) or its derivatives and oxygen donor ligands have been synthesized and structurally characterized (Peng *et al.* 2008; Harvey *et al.* 2008; Kruszynski *et al.* 2009). Recently, we obtained the title cadmium(II) complex containing two different kinds of chelating ligands, by the reaction of cadmium acetate and 5-nitro-1,10-phenanthroline (5NO2phen) in methanol/water mixtures.

The crystal structure of the title compound consists of dimeric units, made up of two Cd cations, two 5NO2phen molecules and four acetate anions (Fig. 1). Two carboxylate groups of acetate anions act as bidentate, and two as monodentate bridging and bidentate chelating ligands. Each Cd atom, in a uncommon seven-coordination environment, is chelated by the two N atoms of 5NOphen and five carbonyl oxygen atoms of three acetate anions. One carboxylate group acts as bidentate, and two as monodentate bridging and bidentate chelating ligands. All coordinating groups are bonded unsymmetrically to the central atom. The Cd—O bridging interactions form a four-membered Cd_2_O_2_ quadrilateral with a Cd—Cd separation of 3.7515 (5) Å.

The dimer crystal structure is stabilized by π-π stacking interactions between adjacent phen rings, with a centroid-centroid distance between *Cg*1(N1/C7—C11) and *Cg4* (C4—C7C11C12) is 3.491 (2) Å (Fig.2). Another intermoleclular π-π stacking interaction exists with a centroid-centroid distance of 3.598 (2) Å between *Cg*2 (N2/C1—C4/C13) and *Cg3* (N4/C13—C16/C24)[symmetry code: - *x*, -*y*, 1 - *z*], which provide additional stabilization to the crystalline networks. There are no classical hydrogen bonding interactions present in the structure.

### Experimental

A solution (6 ml) of methnol containing 5-nitro-1,10-phenanthroline (0.1130 g) was added slowly to a aqueous solution (10 ml) containing cadmium acetate dihydrate (0.1627 g). Block-like single crystals were obtained by slow evaporation of the mixture at room temperature after one month.

### Refinement

The carbon-bound H atoms were placed in calculated positions and were included in the refinement in the riding model approximation, with C—H = 0.93 Å, *U_iso_*(H) = 1.2*U_eq_*(C aromatic) and C—H = 0.96 Å, *U_iso_*(H) = 1.5*U_eq_*(C methyl), respectively. The contrast between the heavier Cd centre and much lighter coordinated O's reflected in unusually large differences in the Hirshfeld Test.

### Crystal data

**Table:**

[Cd_2_(C_2_H_3_O_2_)_4_(C_12_H_7_N_3_O_2_)_2_]	*F*(000) = 1808
*M**_r_* = 911.39	*D*_x_ = 1.870 Mg m^−^^3^
Monoclinic, *P*2_1_/*c*	Mo *K*α radiation, λ = 0.71073 Å
Hall symbol: -P 2ybc	Cell parameters from 7675 reflections
*a* = 18.653 (3) Å	θ = 2.5–28.0°
*b* = 11.2236 (16) Å	µ = 1.39 mm^−^^1^
*c* = 15.510 (2) Å	*T* = 298 K
β = 94.531 (2)°	Block, colourless
*V* = 3236.9 (8) Å^3^	0.45 × 0.30 × 0.27 mm
*Z* = 4	

### Data collection

**Table:**

Bruker SMART CCD area-detector diffractometer	5988 independent reflections
Radiation source: fine-focus sealed tube	4970 reflections with *I* > 2σ(*I*)
graphite	*R*_int_ = 0.022
phi and ω scans	θ_max_ = 25.5°, θ_min_ = 2.2°
Absorption correction: multi-scan (*SADABS*; Bruker, 1997)	*h* = −22→22
*T*_min_ = 0.573, *T*_max_ = 0.705	*k* = −13→12
17895 measured reflections	*l* = −18→18

### Refinement

**Table:**

Refinement on *F*^2^	Primary atom site location: structure-invariant direct methods
Least-squares matrix: full	Secondary atom site location: difference Fourier map
*R*[*F*^2^ > 2σ(*F*^2^)] = 0.025	Hydrogen site location: inferred from neighbouring sites
*wR*(*F*^2^) = 0.057	H-atom parameters constrained
*S* = 1.02	*w* = 1/[σ^2^(*F*_o_^2^) + (0.0212*P*)^2^ + 2.6794*P*] where *P* = (*F*_o_^2^ + 2*F*_c_^2^)/3
5988 reflections	(Δ/σ)_max_ = 0.001
473 parameters	Δρ_max_ = 0.53 e Å^−^^3^
0 restraints	Δρ_min_ = −0.36 e Å^−^^3^

### Special details

**Table:**

Geometry. All e.s.d.'s (except the e.s.d. in the dihedral angle between two l.s. planes)are estimated using the full covariance matrix. The cell e.s.d.'s are takeninto account individually in the estimation of e.s.d.'s in distances, anglesand torsion angles; correlations between e.s.d.'s in cell parameters are onlyused when they are defined by crystal symmetry. An approximate (isotropic)treatment of cell e.s.d.'s is used for estimating e.s.d.'s involving l.s. planes.
Refinement. Refinement of *F*^2^ against ALL reflections. The weighted *R*-factor *wR* and goodness of fit *S* are based on *F*^2^, conventional *R*-factors *R* are based on *F*, with *F* set to zero for negative *F*^2^. The threshold expression of *F*^2^ > σ(*F*^2^) is used only for calculating *R*-factors(gt) *etc*. and is not relevant to the choice of reflections for refinement. *R*-factors based on *F*^2^ are statistically about twice as large as those based on *F*, and *R*- factors based on ALL data will be even larger.

### Fractional atomic coordinates and isotropic or equivalent isotropic displacement parameters (Å^2^)

**Table:**

	*x*	*y*	*z*	*U*_iso_*/*U*_eq_	
Cd1	0.153577 (11)	0.216216 (18)	0.355331 (13)	0.03362 (6)	
Cd2	0.338957 (11)	0.11507 (2)	0.311746 (14)	0.03789 (7)	
O1	0.09881 (18)	−0.2330 (3)	0.7358 (2)	0.0970 (11)	
O2	0.20827 (17)	−0.1747 (3)	0.73914 (19)	0.0898 (10)	
O3	0.3288 (2)	−0.3161 (3)	0.6785 (2)	0.1007 (11)	
O4	0.38206 (17)	−0.2012 (3)	0.77268 (17)	0.0841 (9)	
O5	0.06501 (14)	0.3349 (2)	0.42283 (18)	0.0681 (7)	
O6	0.13052 (13)	0.4173 (2)	0.33063 (17)	0.0642 (7)	
O7	0.12037 (12)	0.1684 (2)	0.20738 (14)	0.0525 (6)	
O8	0.21683 (11)	0.0811 (2)	0.26526 (13)	0.0483 (6)	
O9	0.27715 (11)	0.27093 (18)	0.36736 (15)	0.0481 (5)	
O10	0.37470 (13)	0.3381 (2)	0.31632 (16)	0.0600 (6)	
O11	0.35271 (13)	0.1120 (2)	0.15096 (17)	0.0677 (7)	
O12	0.42983 (13)	0.0344 (3)	0.24598 (15)	0.0675 (7)	
N1	0.08544 (11)	0.0405 (2)	0.38311 (14)	0.0332 (5)	
N2	0.18945 (11)	0.1377 (2)	0.49403 (14)	0.0312 (5)	
N3	0.14639 (18)	−0.1784 (3)	0.70622 (19)	0.0553 (7)	
N4	0.31140 (12)	−0.0453 (2)	0.40541 (15)	0.0358 (6)	
N5	0.41221 (12)	0.1226 (2)	0.44876 (16)	0.0389 (6)	
N6	0.35865 (17)	−0.2219 (3)	0.6989 (2)	0.0574 (8)	
C1	0.23903 (15)	0.1877 (3)	0.54803 (19)	0.0372 (7)	
H1	0.2637	0.2534	0.5291	0.045*	
C2	0.25597 (16)	0.1468 (3)	0.63140 (19)	0.0419 (7)	
H2	0.2903	0.1861	0.6677	0.050*	
C3	0.22194 (16)	0.0487 (3)	0.65971 (19)	0.0415 (7)	
H3A	0.2325	0.0211	0.7158	0.050*	
C4	0.17054 (14)	−0.0108 (3)	0.60346 (17)	0.0330 (6)	
C5	0.13060 (16)	−0.1160 (3)	0.62330 (19)	0.0387 (7)	
C6	0.07871 (16)	−0.1638 (3)	0.5686 (2)	0.0406 (7)	
H6	0.0536	−0.2305	0.5854	0.049*	
C7	0.06202 (14)	−0.1134 (2)	0.48562 (19)	0.0346 (6)	
C8	0.00779 (15)	−0.1598 (3)	0.4266 (2)	0.0413 (7)	
H8	−0.0187	−0.2258	0.4413	0.050*	
C9	−0.00546 (15)	−0.1073 (3)	0.3480 (2)	0.0446 (8)	
H9	−0.0405	−0.1373	0.3079	0.054*	
C10	0.03473 (15)	−0.0076 (3)	0.32916 (19)	0.0409 (7)	
H10	0.0254	0.0277	0.2752	0.049*	
C11	0.09979 (13)	−0.0126 (2)	0.46114 (17)	0.0294 (6)	
C12	0.15494 (13)	0.0393 (2)	0.52054 (17)	0.0284 (6)	
C13	0.26377 (17)	−0.1284 (3)	0.3811 (2)	0.0444 (8)	
H13	0.2411	−0.1253	0.3255	0.053*	
C14	0.24642 (18)	−0.2205 (3)	0.4360 (2)	0.0497 (8)	
H14	0.2127	−0.2777	0.4172	0.060*	
C15	0.27917 (18)	−0.2260 (3)	0.5171 (2)	0.0479 (8)	
H15	0.2676	−0.2873	0.5539	0.057*	
C16	0.33037 (15)	−0.1403 (3)	0.54626 (19)	0.0368 (7)	
C17	0.36932 (16)	−0.1328 (3)	0.63118 (19)	0.0407 (7)	
C18	0.41653 (16)	−0.0459 (3)	0.6530 (2)	0.0468 (8)	
H18	0.4386	−0.0434	0.7088	0.056*	
C19	0.43346 (15)	0.0422 (3)	0.5924 (2)	0.0419 (7)	
C20	0.48556 (17)	0.1302 (3)	0.6119 (2)	0.0522 (9)	
H20	0.5096	0.1340	0.6667	0.063*	
C21	0.50047 (18)	0.2098 (3)	0.5500 (2)	0.0544 (9)	
H21	0.5357	0.2676	0.5613	0.065*	
C22	0.46233 (16)	0.2035 (3)	0.4695 (2)	0.0487 (8)	
H22	0.4726	0.2592	0.4279	0.058*	
C23	0.39777 (14)	0.0407 (2)	0.50929 (18)	0.0336 (6)	
C24	0.34502 (14)	−0.0502 (2)	0.48610 (18)	0.0327 (6)	
C25	0.08465 (16)	0.4241 (3)	0.3849 (2)	0.0400 (7)	
C26	0.05399 (19)	0.5449 (3)	0.4043 (2)	0.0534 (9)	
H26A	0.0024	0.5415	0.3971	0.080*	
H26B	0.0711	0.6029	0.3653	0.080*	
H26C	0.0690	0.5670	0.4627	0.080*	
C27	0.17103 (16)	0.0989 (3)	0.20191 (18)	0.0382 (7)	
C28	0.1775 (2)	0.0289 (3)	0.1205 (2)	0.0625 (10)	
H28A	0.1586	−0.0498	0.1274	0.094*	
H28B	0.2271	0.0237	0.1088	0.094*	
H28C	0.1508	0.0680	0.0731	0.094*	
C29	0.32114 (16)	0.3550 (3)	0.35672 (19)	0.0393 (7)	
C30	0.3064 (2)	0.4736 (3)	0.3955 (2)	0.0621 (10)	
H30A	0.3158	0.4695	0.4572	0.093*	
H30B	0.2569	0.4948	0.3816	0.093*	
H30C	0.3369	0.5327	0.3727	0.093*	
C31	0.40692 (16)	0.0530 (3)	0.1693 (2)	0.0455 (8)	
C32	0.4482 (2)	−0.0005 (4)	0.0988 (2)	0.0668 (11)	
H32A	0.4285	−0.0772	0.0831	0.100*	
H32B	0.4979	−0.0093	0.1194	0.100*	
H32C	0.4446	0.0509	0.0492	0.100*	

### Atomic displacement parameters (Å^2^)

**Table:**

	*U*^11^	*U*^22^	*U*^33^	*U*^12^	*U*^13^	*U*^23^
Cd1	0.03478 (11)	0.03301 (12)	0.03320 (12)	0.00200 (9)	0.00347 (8)	0.00199 (9)
Cd2	0.03589 (12)	0.04232 (14)	0.03546 (13)	0.00713 (10)	0.00274 (9)	0.00228 (10)
O1	0.108 (2)	0.112 (3)	0.072 (2)	−0.028 (2)	0.0169 (18)	0.0439 (19)
O2	0.090 (2)	0.093 (2)	0.083 (2)	0.0017 (18)	−0.0210 (17)	0.0416 (18)
O3	0.153 (3)	0.071 (2)	0.076 (2)	−0.020 (2)	−0.004 (2)	0.0205 (17)
O4	0.110 (2)	0.094 (2)	0.0455 (17)	−0.0083 (18)	−0.0104 (16)	0.0207 (15)
O5	0.0853 (19)	0.0397 (14)	0.0835 (19)	0.0032 (13)	0.0332 (15)	0.0058 (13)
O6	0.0615 (15)	0.0558 (16)	0.0793 (18)	0.0120 (12)	0.0319 (14)	0.0035 (13)
O7	0.0557 (14)	0.0577 (15)	0.0440 (13)	0.0169 (12)	0.0033 (11)	0.0046 (11)
O8	0.0389 (11)	0.0693 (16)	0.0357 (12)	0.0023 (11)	−0.0022 (9)	−0.0026 (11)
O9	0.0405 (12)	0.0354 (13)	0.0681 (16)	−0.0020 (10)	0.0028 (11)	0.0037 (11)
O10	0.0537 (14)	0.0607 (16)	0.0675 (17)	0.0029 (12)	0.0158 (12)	0.0133 (13)
O11	0.0544 (15)	0.0778 (19)	0.0709 (18)	0.0270 (14)	0.0054 (13)	0.0039 (14)
O12	0.0626 (15)	0.097 (2)	0.0430 (15)	0.0242 (15)	0.0035 (12)	−0.0009 (14)
N1	0.0309 (12)	0.0358 (14)	0.0329 (13)	−0.0005 (10)	0.0028 (10)	−0.0028 (10)
N2	0.0317 (12)	0.0315 (13)	0.0304 (13)	−0.0011 (10)	0.0025 (10)	−0.0017 (10)
N3	0.068 (2)	0.0501 (18)	0.0484 (18)	0.0061 (16)	0.0112 (16)	0.0147 (14)
N4	0.0377 (13)	0.0329 (14)	0.0361 (14)	0.0031 (11)	−0.0008 (10)	−0.0048 (11)
N5	0.0386 (13)	0.0345 (14)	0.0429 (15)	0.0015 (11)	−0.0015 (11)	0.0004 (11)
N6	0.0659 (19)	0.056 (2)	0.051 (2)	0.0055 (16)	0.0066 (15)	0.0101 (15)
C1	0.0371 (15)	0.0371 (17)	0.0372 (17)	−0.0041 (13)	0.0022 (13)	−0.0056 (13)
C2	0.0416 (16)	0.046 (2)	0.0370 (17)	−0.0005 (14)	−0.0030 (13)	−0.0127 (14)
C3	0.0447 (17)	0.051 (2)	0.0290 (16)	0.0092 (15)	0.0005 (13)	−0.0032 (14)
C4	0.0326 (14)	0.0359 (16)	0.0317 (15)	0.0085 (12)	0.0096 (12)	−0.0012 (12)
C5	0.0446 (17)	0.0372 (17)	0.0360 (17)	0.0099 (14)	0.0137 (13)	0.0094 (13)
C6	0.0396 (16)	0.0326 (17)	0.052 (2)	0.0020 (13)	0.0179 (14)	0.0054 (14)
C7	0.0311 (14)	0.0281 (16)	0.0458 (18)	0.0030 (12)	0.0102 (12)	−0.0037 (13)
C8	0.0318 (15)	0.0306 (17)	0.063 (2)	−0.0027 (13)	0.0110 (14)	−0.0094 (15)
C9	0.0352 (16)	0.0437 (19)	0.054 (2)	−0.0011 (14)	−0.0021 (14)	−0.0171 (16)
C10	0.0379 (16)	0.0472 (19)	0.0367 (17)	−0.0010 (14)	−0.0018 (13)	−0.0067 (14)
C11	0.0255 (13)	0.0278 (15)	0.0354 (16)	0.0037 (11)	0.0063 (11)	−0.0040 (12)
C12	0.0264 (13)	0.0300 (15)	0.0294 (14)	0.0040 (11)	0.0053 (11)	−0.0005 (11)
C13	0.0512 (18)	0.0377 (19)	0.0435 (19)	0.0017 (15)	−0.0005 (14)	−0.0115 (14)
C14	0.0525 (19)	0.0393 (19)	0.057 (2)	−0.0088 (15)	0.0051 (16)	−0.0115 (16)
C15	0.055 (2)	0.0375 (19)	0.053 (2)	0.0018 (15)	0.0137 (16)	0.0038 (15)
C16	0.0368 (15)	0.0343 (17)	0.0401 (17)	0.0089 (13)	0.0071 (13)	−0.0015 (13)
C17	0.0456 (17)	0.0437 (19)	0.0333 (16)	0.0118 (15)	0.0058 (13)	0.0073 (14)
C18	0.0456 (18)	0.058 (2)	0.0352 (18)	0.0124 (16)	−0.0049 (14)	−0.0001 (15)
C19	0.0371 (16)	0.0456 (19)	0.0421 (18)	0.0085 (14)	−0.0029 (13)	−0.0019 (14)
C20	0.0439 (18)	0.054 (2)	0.056 (2)	0.0041 (16)	−0.0159 (16)	−0.0109 (17)
C21	0.0441 (18)	0.043 (2)	0.073 (3)	−0.0032 (15)	−0.0115 (17)	−0.0049 (18)
C22	0.0423 (17)	0.0399 (19)	0.063 (2)	−0.0012 (15)	−0.0023 (16)	0.0036 (16)
C23	0.0269 (14)	0.0356 (17)	0.0379 (16)	0.0102 (12)	−0.0004 (12)	−0.0039 (13)
C24	0.0315 (14)	0.0331 (16)	0.0341 (16)	0.0103 (12)	0.0057 (12)	−0.0010 (12)
C25	0.0413 (17)	0.0392 (19)	0.0387 (17)	0.0038 (14)	−0.0021 (14)	−0.0027 (14)
C26	0.068 (2)	0.043 (2)	0.051 (2)	0.0145 (17)	0.0136 (17)	−0.0001 (16)
C27	0.0400 (16)	0.0423 (19)	0.0325 (16)	−0.0068 (14)	0.0047 (13)	0.0020 (13)
C28	0.070 (2)	0.070 (3)	0.046 (2)	0.001 (2)	−0.0017 (18)	−0.0157 (18)
C29	0.0422 (17)	0.0385 (18)	0.0357 (17)	−0.0014 (14)	−0.0054 (14)	0.0044 (13)
C30	0.079 (3)	0.041 (2)	0.065 (2)	−0.0060 (18)	−0.006 (2)	−0.0071 (17)
C31	0.0398 (17)	0.050 (2)	0.048 (2)	0.0037 (15)	0.0097 (15)	0.0002 (16)
C32	0.070 (2)	0.076 (3)	0.057 (2)	0.013 (2)	0.0203 (19)	−0.005 (2)

### Geometric parameters (Å, °)

**Table:**

Cd1—O6	2.324 (2)	C5—C6	1.347 (4)
Cd1—N2	2.372 (2)	C6—C7	1.419 (4)
Cd1—O9	2.378 (2)	C6—H6	0.9300
Cd1—O7	2.390 (2)	C7—C11	1.401 (4)
Cd1—N1	2.403 (2)	C7—C8	1.410 (4)
Cd1—O5	2.423 (2)	C8—C9	1.358 (4)
Cd1—O8	2.429 (2)	C8—H8	0.9300
Cd1—C25	2.721 (3)	C9—C10	1.391 (4)
Cd2—O12	2.238 (2)	C9—H9	0.9300
Cd2—O9	2.300 (2)	C10—H10	0.9300
Cd2—O8	2.365 (2)	C11—C12	1.448 (4)
Cd2—N4	2.394 (2)	C13—C14	1.393 (4)
Cd2—N5	2.435 (2)	C13—H13	0.9300
Cd2—O11	2.527 (3)	C14—C15	1.355 (5)
Cd2—O10	2.590 (3)	C14—H14	0.9300
Cd2—C31	2.723 (3)	C15—C16	1.405 (4)
O1—N3	1.199 (4)	C15—H15	0.9300
O2—N3	1.225 (4)	C16—C24	1.417 (4)
O3—N6	1.224 (4)	C16—C17	1.455 (4)
O4—N6	1.215 (4)	C17—C18	1.339 (4)
O5—C25	1.232 (4)	C18—C19	1.416 (4)
O6—C25	1.249 (4)	C18—H18	0.9300
O7—C27	1.233 (4)	C19—C20	1.402 (4)
O8—C27	1.266 (3)	C19—C23	1.404 (4)
O9—C29	1.270 (3)	C20—C21	1.356 (5)
O10—C29	1.235 (4)	C20—H20	0.9300
O11—C31	1.224 (4)	C21—C22	1.389 (5)
O12—C31	1.248 (4)	C21—H21	0.9300
N1—C10	1.327 (3)	C22—H22	0.9300
N1—C11	1.356 (3)	C23—C24	1.443 (4)
N2—C1	1.323 (3)	C25—C26	1.510 (4)
N2—C12	1.358 (3)	C26—H26A	0.9600
N3—C5	1.473 (4)	C26—H26B	0.9600
N4—C13	1.322 (4)	C26—H26C	0.9600
N4—C24	1.356 (3)	C27—C28	1.501 (4)
N5—C22	1.325 (4)	C28—H28A	0.9600
N5—C23	1.355 (4)	C28—H28B	0.9600
N6—C17	1.475 (4)	C28—H28C	0.9600
C1—C2	1.385 (4)	C29—C30	1.495 (4)
C1—H1	0.9300	C30—H30A	0.9600
C2—C3	1.361 (4)	C30—H30B	0.9600
C2—H2	0.9300	C30—H30C	0.9600
C3—C4	1.413 (4)	C31—C32	1.511 (4)
C3—H3A	0.9300	C32—H32A	0.9600
C4—C12	1.413 (4)	C32—H32B	0.9600
C4—C5	1.442 (4)	C32—H32C	0.9600
			
O6—Cd1—N2	123.08 (9)	C4—C5—N3	120.3 (3)
O6—Cd1—O9	85.86 (8)	C5—C6—C7	120.5 (3)
N2—Cd1—O9	79.78 (8)	C5—C6—H6	119.7
O6—Cd1—O7	91.67 (9)	C7—C6—H6	119.7
N2—Cd1—O7	145.14 (8)	C11—C7—C8	118.2 (3)
O9—Cd1—O7	108.02 (8)	C11—C7—C6	119.3 (3)
O6—Cd1—N1	137.13 (8)	C8—C7—C6	122.5 (3)
N2—Cd1—N1	69.37 (7)	C9—C8—C7	119.6 (3)
O9—Cd1—N1	136.12 (7)	C9—C8—H8	120.2
O7—Cd1—N1	83.53 (8)	C7—C8—H8	120.2
O6—Cd1—O5	54.11 (8)	C8—C9—C10	118.3 (3)
N2—Cd1—O5	88.02 (8)	C8—C9—H9	120.8
O9—Cd1—O5	120.91 (8)	C10—C9—H9	120.8
O7—Cd1—O5	113.85 (9)	N1—C10—C9	124.3 (3)
N1—Cd1—O5	89.16 (8)	N1—C10—H10	117.8
O6—Cd1—O8	127.10 (8)	C9—C10—H10	117.8
N2—Cd1—O8	100.06 (7)	N1—C11—C7	121.9 (2)
O9—Cd1—O8	72.22 (7)	N1—C11—C12	118.1 (2)
O7—Cd1—O8	53.74 (7)	C7—C11—C12	120.0 (3)
N1—Cd1—O8	83.04 (7)	N2—C12—C4	122.1 (2)
O5—Cd1—O8	165.93 (8)	N2—C12—C11	117.7 (2)
O6—Cd1—C25	27.24 (8)	C4—C12—C11	120.3 (2)
N2—Cd1—C25	105.62 (8)	N4—C13—C14	122.1 (3)
O9—Cd1—C25	103.49 (8)	N4—C13—H13	118.9
O7—Cd1—C25	105.32 (8)	C14—C13—H13	118.9
N1—Cd1—C25	114.20 (9)	C15—C14—C13	119.4 (3)
O5—Cd1—C25	26.93 (8)	C15—C14—H14	120.3
O8—Cd1—C25	152.81 (8)	C13—C14—H14	120.3
O12—Cd2—O9	154.02 (9)	C14—C15—C16	120.8 (3)
O12—Cd2—O8	122.81 (8)	C14—C15—H15	119.6
O9—Cd2—O8	74.79 (8)	C16—C15—H15	119.6
O12—Cd2—N4	100.31 (9)	C15—C16—C24	116.0 (3)
O9—Cd2—N4	101.90 (8)	C15—C16—C17	127.5 (3)
O8—Cd2—N4	79.41 (8)	C24—C16—C17	116.5 (3)
O12—Cd2—N5	91.08 (8)	C18—C17—C16	122.8 (3)
O9—Cd2—N5	84.82 (8)	C18—C17—N6	115.9 (3)
O8—Cd2—N5	137.05 (8)	C16—C17—N6	121.3 (3)
N4—Cd2—N5	68.23 (8)	C17—C18—C19	121.2 (3)
O12—Cd2—O11	53.49 (8)	C17—C18—H18	119.4
O9—Cd2—O11	118.25 (8)	C19—C18—H18	119.4
O8—Cd2—O11	82.38 (8)	C20—C19—C23	118.4 (3)
N4—Cd2—O11	129.19 (8)	C20—C19—C18	122.4 (3)
N5—Cd2—O11	140.16 (8)	C23—C19—C18	119.1 (3)
O12—Cd2—O10	101.60 (9)	C21—C20—C19	119.3 (3)
O9—Cd2—O10	52.43 (7)	C21—C20—H20	120.4
O8—Cd2—O10	113.80 (8)	C19—C20—H20	120.4
N4—Cd2—O10	141.00 (8)	C20—C21—C22	118.9 (3)
N5—Cd2—O10	79.47 (8)	C20—C21—H21	120.5
O11—Cd2—O10	89.67 (8)	C22—C21—H21	120.5
O12—Cd2—C31	26.95 (8)	N5—C22—C21	123.7 (3)
O9—Cd2—C31	141.09 (9)	N5—C22—H22	118.1
O8—Cd2—C31	102.15 (8)	C21—C22—H22	118.1
N4—Cd2—C31	115.84 (9)	N5—C23—C19	121.5 (3)
N5—Cd2—C31	116.85 (9)	N5—C23—C24	118.4 (2)
O11—Cd2—C31	26.62 (8)	C19—C23—C24	120.1 (3)
O10—Cd2—C31	97.71 (9)	N4—C24—C16	122.5 (3)
C25—O5—Cd1	90.12 (19)	N4—C24—C23	117.3 (3)
C25—O6—Cd1	94.4 (2)	C16—C24—C23	120.2 (3)
C27—O7—Cd1	93.79 (18)	O5—C25—O6	121.1 (3)
C27—O8—Cd2	141.80 (19)	O5—C25—C26	120.2 (3)
C27—O8—Cd1	91.11 (18)	O6—C25—C26	118.7 (3)
Cd2—O8—Cd1	102.95 (8)	O5—C25—Cd1	62.96 (17)
C29—O9—Cd2	99.70 (19)	O6—C25—Cd1	58.39 (17)
C29—O9—Cd1	144.47 (19)	C26—C25—Cd1	174.0 (2)
Cd2—O9—Cd1	106.59 (8)	C25—C26—H26A	109.5
C29—O10—Cd2	86.91 (19)	C25—C26—H26B	109.5
C31—O11—Cd2	85.6 (2)	H26A—C26—H26B	109.5
C31—O12—Cd2	98.7 (2)	C25—C26—H26C	109.5
C10—N1—C11	117.7 (2)	H26A—C26—H26C	109.5
C10—N1—Cd1	125.5 (2)	H26B—C26—H26C	109.5
C11—N1—Cd1	116.77 (17)	O7—C27—O8	121.3 (3)
C1—N2—C12	118.5 (2)	O7—C27—C28	120.1 (3)
C1—N2—Cd1	123.35 (19)	O8—C27—C28	118.5 (3)
C12—N2—Cd1	118.05 (16)	C27—C28—H28A	109.5
O1—N3—O2	124.0 (3)	C27—C28—H28B	109.5
O1—N3—C5	118.3 (3)	H28A—C28—H28B	109.5
O2—N3—C5	117.7 (3)	C27—C28—H28C	109.5
C13—N4—C24	119.1 (3)	H28A—C28—H28C	109.5
C13—N4—Cd2	121.9 (2)	H28B—C28—H28C	109.5
C24—N4—Cd2	118.97 (18)	O10—C29—O9	120.5 (3)
C22—N5—C23	118.1 (3)	O10—C29—C30	121.6 (3)
C22—N5—Cd2	124.9 (2)	O9—C29—C30	117.9 (3)
C23—N5—Cd2	116.91 (17)	C29—C30—H30A	109.5
O4—N6—O3	122.0 (3)	C29—C30—H30B	109.5
O4—N6—C17	118.8 (3)	H30A—C30—H30B	109.5
O3—N6—C17	119.1 (3)	C29—C30—H30C	109.5
N2—C1—C2	123.2 (3)	H30A—C30—H30C	109.5
N2—C1—H1	118.4	H30B—C30—H30C	109.5
C2—C1—H1	118.4	O11—C31—O12	121.8 (3)
C3—C2—C1	119.4 (3)	O11—C31—C32	120.4 (3)
C3—C2—H2	120.3	O12—C31—C32	117.8 (3)
C1—C2—H2	120.3	O11—C31—Cd2	67.75 (19)
C2—C3—C4	119.7 (3)	O12—C31—Cd2	54.32 (16)
C2—C3—H3A	120.2	C32—C31—Cd2	170.2 (3)
C4—C3—H3A	120.2	C31—C32—H32A	109.5
C3—C4—C12	117.0 (3)	C31—C32—H32B	109.5
C3—C4—C5	126.3 (3)	H32A—C32—H32B	109.5
C12—C4—C5	116.6 (3)	C31—C32—H32C	109.5
C6—C5—C4	123.3 (3)	H32A—C32—H32C	109.5
C6—C5—N3	116.4 (3)	H32B—C32—H32C	109.5
			
O6—Cd1—O5—C25	−2.85 (18)	O10—Cd2—N5—C22	−23.4 (2)
N2—Cd1—O5—C25	131.5 (2)	C31—Cd2—N5—C22	70.0 (3)
O9—Cd1—O5—C25	54.7 (2)	O12—Cd2—N5—C23	−105.1 (2)
O7—Cd1—O5—C25	−76.6 (2)	O9—Cd2—N5—C23	100.6 (2)
N1—Cd1—O5—C25	−159.1 (2)	O8—Cd2—N5—C23	39.6 (2)
O8—Cd1—O5—C25	−103.0 (3)	N4—Cd2—N5—C23	−4.41 (18)
N2—Cd1—O6—C25	−55.7 (2)	O11—Cd2—N5—C23	−130.10 (19)
O9—Cd1—O6—C25	−130.7 (2)	O10—Cd2—N5—C23	153.3 (2)
O7—Cd1—O6—C25	121.4 (2)	C31—Cd2—N5—C23	−113.3 (2)
N1—Cd1—O6—C25	39.1 (3)	C12—N2—C1—C2	−2.1 (4)
O5—Cd1—O6—C25	2.82 (18)	Cd1—N2—C1—C2	174.9 (2)
O8—Cd1—O6—C25	165.36 (17)	N2—C1—C2—C3	1.8 (5)
O6—Cd1—O7—C27	137.37 (19)	C1—C2—C3—C4	0.8 (4)
N2—Cd1—O7—C27	−46.9 (2)	C2—C3—C4—C12	−2.8 (4)
O9—Cd1—O7—C27	51.2 (2)	C2—C3—C4—C5	179.3 (3)
N1—Cd1—O7—C27	−85.36 (19)	C3—C4—C5—C6	175.3 (3)
O5—Cd1—O7—C27	−171.56 (18)	C12—C4—C5—C6	−2.6 (4)
O8—Cd1—O7—C27	0.73 (17)	C3—C4—C5—N3	−5.7 (4)
C25—Cd1—O7—C27	161.26 (18)	C12—C4—C5—N3	176.4 (2)
O12—Cd2—O8—C27	−64.1 (4)	O1—N3—C5—C6	−29.0 (5)
O9—Cd2—O8—C27	94.8 (3)	O2—N3—C5—C6	148.4 (3)
N4—Cd2—O8—C27	−159.6 (3)	O1—N3—C5—C4	152.0 (3)
N5—Cd2—O8—C27	159.4 (3)	O2—N3—C5—C4	−30.6 (4)
O11—Cd2—O8—C27	−27.3 (3)	C4—C5—C6—C7	1.9 (4)
O10—Cd2—O8—C27	59.0 (3)	N3—C5—C6—C7	−177.1 (3)
C31—Cd2—O8—C27	−45.2 (3)	C5—C6—C7—C11	−0.4 (4)
O12—Cd2—O8—Cd1	−173.05 (9)	C5—C6—C7—C8	−179.5 (3)
O9—Cd2—O8—Cd1	−14.12 (8)	C11—C7—C8—C9	1.0 (4)
N4—Cd2—O8—Cd1	91.44 (9)	C6—C7—C8—C9	−179.9 (3)
N5—Cd2—O8—Cd1	50.45 (14)	C7—C8—C9—C10	−1.0 (4)
O11—Cd2—O8—Cd1	−136.21 (9)	C11—N1—C10—C9	1.2 (4)
O10—Cd2—O8—Cd1	−49.96 (10)	Cd1—N1—C10—C9	−177.9 (2)
C31—Cd2—O8—Cd1	−154.15 (9)	C8—C9—C10—N1	−0.2 (5)
O6—Cd1—O8—C27	−60.1 (2)	C10—N1—C11—C7	−1.2 (4)
N2—Cd1—O8—C27	153.89 (17)	Cd1—N1—C11—C7	178.05 (19)
O9—Cd1—O8—C27	−130.36 (19)	C10—N1—C11—C12	−179.9 (2)
O7—Cd1—O8—C27	−0.71 (16)	Cd1—N1—C11—C12	−0.7 (3)
N1—Cd1—O8—C27	86.35 (18)	C8—C7—C11—N1	0.1 (4)
O5—Cd1—O8—C27	29.6 (4)	C6—C7—C11—N1	−179.0 (2)
C25—Cd1—O8—C27	−45.4 (3)	C8—C7—C11—C12	178.8 (2)
O6—Cd1—O8—Cd2	84.10 (11)	C6—C7—C11—C12	−0.3 (4)
N2—Cd1—O8—Cd2	−61.92 (9)	C1—N2—C12—C4	−0.1 (4)
O9—Cd1—O8—Cd2	13.83 (7)	Cd1—N2—C12—C4	−177.31 (19)
O7—Cd1—O8—Cd2	143.48 (12)	C1—N2—C12—C11	178.7 (2)
N1—Cd1—O8—Cd2	−129.46 (9)	Cd1—N2—C12—C11	1.6 (3)
O5—Cd1—O8—Cd2	173.8 (3)	C3—C4—C12—N2	2.5 (4)
C25—Cd1—O8—Cd2	98.76 (17)	C5—C4—C12—N2	−179.4 (2)
O12—Cd2—O9—C29	−4.8 (3)	C3—C4—C12—C11	−176.3 (2)
O8—Cd2—O9—C29	−141.16 (19)	C5—C4—C12—C11	1.8 (4)
N4—Cd2—O9—C29	143.44 (18)	N1—C11—C12—N2	−0.6 (4)
N5—Cd2—O9—C29	77.00 (18)	C7—C11—C12—N2	−179.3 (2)
O11—Cd2—O9—C29	−68.74 (19)	N1—C11—C12—C4	178.3 (2)
O10—Cd2—O9—C29	−3.69 (16)	C7—C11—C12—C4	−0.4 (4)
C31—Cd2—O9—C29	−50.6 (2)	C24—N4—C13—C14	0.6 (4)
O12—Cd2—O9—Cd1	151.05 (15)	Cd2—N4—C13—C14	−178.6 (2)
O8—Cd2—O9—Cd1	14.68 (8)	N4—C13—C14—C15	0.0 (5)
N4—Cd2—O9—Cd1	−60.72 (10)	C13—C14—C15—C16	−0.2 (5)
N5—Cd2—O9—Cd1	−127.16 (10)	C14—C15—C16—C24	−0.1 (4)
O11—Cd2—O9—Cd1	87.09 (11)	C14—C15—C16—C17	179.1 (3)
O10—Cd2—O9—Cd1	152.15 (13)	C15—C16—C17—C18	−179.0 (3)
C31—Cd2—O9—Cd1	105.27 (13)	C24—C16—C17—C18	0.3 (4)
O6—Cd1—O9—C29	−9.6 (4)	C15—C16—C17—N6	1.0 (5)
N2—Cd1—O9—C29	−134.3 (4)	C24—C16—C17—N6	−179.8 (3)
O7—Cd1—O9—C29	80.8 (4)	O4—N6—C17—C18	12.9 (5)
N1—Cd1—O9—C29	−179.6 (3)	O3—N6—C17—C18	−164.6 (3)
O5—Cd1—O9—C29	−52.9 (4)	O4—N6—C17—C16	−167.1 (3)
O8—Cd1—O9—C29	121.5 (4)	O3—N6—C17—C16	15.5 (5)
C25—Cd1—O9—C29	−30.5 (4)	C16—C17—C18—C19	−2.5 (5)
O6—Cd1—O9—Cd2	−145.66 (10)	N6—C17—C18—C19	177.5 (3)
N2—Cd1—O9—Cd2	89.65 (9)	C17—C18—C19—C20	−176.4 (3)
O7—Cd1—O9—Cd2	−55.24 (10)	C17—C18—C19—C23	2.6 (4)
N1—Cd1—O9—Cd2	44.39 (15)	C23—C19—C20—C21	−0.8 (5)
O5—Cd1—O9—Cd2	171.10 (8)	C18—C19—C20—C21	178.1 (3)
O8—Cd1—O9—Cd2	−14.48 (8)	C19—C20—C21—C22	1.6 (5)
C25—Cd1—O9—Cd2	−166.57 (9)	C23—N5—C22—C21	−0.6 (5)
O12—Cd2—O10—C29	−176.75 (18)	Cd2—N5—C22—C21	176.1 (2)
O9—Cd2—O10—C29	3.74 (17)	C20—C21—C22—N5	−0.9 (5)
O8—Cd2—O10—C29	49.21 (19)	C22—N5—C23—C19	1.3 (4)
N4—Cd2—O10—C29	−53.8 (2)	Cd2—N5—C23—C19	−175.7 (2)
N5—Cd2—O10—C29	−87.82 (18)	C22—N5—C23—C24	−177.9 (3)
O11—Cd2—O10—C29	130.73 (18)	Cd2—N5—C23—C24	5.2 (3)
C31—Cd2—O10—C29	156.19 (18)	C20—C19—C23—N5	−0.6 (4)
O12—Cd2—O11—C31	3.4 (2)	C18—C19—C23—N5	−179.6 (3)
O9—Cd2—O11—C31	154.07 (19)	C20—C19—C23—C24	178.5 (3)
O8—Cd2—O11—C31	−137.8 (2)	C18—C19—C23—C24	−0.5 (4)
N4—Cd2—O11—C31	−68.2 (2)	C13—N4—C24—C16	−0.9 (4)
N5—Cd2—O11—C31	35.1 (3)	Cd2—N4—C24—C16	178.31 (19)
O10—Cd2—O11—C31	108.1 (2)	C13—N4—C24—C23	178.7 (2)
O9—Cd2—O12—C31	−83.3 (3)	Cd2—N4—C24—C23	−2.1 (3)
O8—Cd2—O12—C31	44.3 (3)	C15—C16—C24—N4	0.7 (4)
N4—Cd2—O12—C31	128.3 (2)	C17—C16—C24—N4	−178.6 (2)
N5—Cd2—O12—C31	−163.7 (2)	C15—C16—C24—C23	−178.9 (3)
O11—Cd2—O12—C31	−3.3 (2)	C17—C16—C24—C23	1.8 (4)
O10—Cd2—O12—C31	−84.2 (2)	N5—C23—C24—N4	−2.1 (4)
O6—Cd1—N1—C10	63.3 (3)	C19—C23—C24—N4	178.7 (2)
N2—Cd1—N1—C10	−179.8 (2)	N5—C23—C24—C16	177.5 (2)
O9—Cd1—N1—C10	−131.5 (2)	C19—C23—C24—C16	−1.7 (4)
O7—Cd1—N1—C10	−22.1 (2)	Cd1—O5—C25—O6	5.0 (3)
O5—Cd1—N1—C10	92.0 (2)	Cd1—O5—C25—C26	−174.2 (3)
O8—Cd1—N1—C10	−76.3 (2)	Cd1—O6—C25—O5	−5.3 (3)
C25—Cd1—N1—C10	81.8 (2)	Cd1—O6—C25—C26	174.0 (2)
O6—Cd1—N1—C11	−115.82 (19)	O6—Cd1—C25—O5	175.0 (3)
N2—Cd1—N1—C11	1.04 (17)	N2—Cd1—C25—O5	−51.0 (2)
O9—Cd1—N1—C11	49.4 (2)	O9—Cd1—C25—O5	−134.0 (2)
O7—Cd1—N1—C11	158.71 (19)	O7—Cd1—C25—O5	112.7 (2)
O5—Cd1—N1—C11	−87.17 (19)	N1—Cd1—C25—O5	23.0 (2)
O8—Cd1—N1—C11	104.57 (18)	O8—Cd1—C25—O5	148.8 (2)
C25—Cd1—N1—C11	−97.37 (19)	N2—Cd1—C25—O6	134.05 (19)
O6—Cd1—N2—C1	−44.8 (2)	O9—Cd1—C25—O6	51.1 (2)
O9—Cd1—N2—C1	33.4 (2)	O7—Cd1—C25—O6	−62.2 (2)
O7—Cd1—N2—C1	140.3 (2)	N1—Cd1—C25—O6	−151.94 (19)
N1—Cd1—N2—C1	−178.4 (2)	O5—Cd1—C25—O6	−175.0 (3)
O5—Cd1—N2—C1	−88.5 (2)	O8—Cd1—C25—O6	−26.2 (3)
O8—Cd1—N2—C1	103.1 (2)	Cd1—O7—C27—O8	−1.3 (3)
C25—Cd1—N2—C1	−67.9 (2)	Cd1—O7—C27—C28	175.7 (3)
O6—Cd1—N2—C12	132.22 (18)	Cd2—O8—C27—O7	−111.5 (3)
O9—Cd1—N2—C12	−149.61 (19)	Cd1—O8—C27—O7	1.3 (3)
O7—Cd1—N2—C12	−42.7 (2)	Cd2—O8—C27—C28	71.4 (4)
N1—Cd1—N2—C12	−1.36 (17)	Cd1—O8—C27—C28	−175.8 (3)
O5—Cd1—N2—C12	88.49 (19)	Cd2—O10—C29—O9	−6.2 (3)
O8—Cd1—N2—C12	−79.92 (19)	Cd2—O10—C29—C30	172.9 (3)
C25—Cd1—N2—C12	109.10 (19)	Cd2—O9—C29—O10	7.1 (3)
O12—Cd2—N4—C13	−90.4 (2)	Cd1—O9—C29—O10	−130.4 (3)
O9—Cd2—N4—C13	103.1 (2)	Cd2—O9—C29—C30	−172.1 (2)
O8—Cd2—N4—C13	31.3 (2)	Cd1—O9—C29—C30	50.4 (5)
N5—Cd2—N4—C13	−177.4 (2)	Cd2—O11—C31—O12	−5.7 (3)
O11—Cd2—N4—C13	−39.6 (3)	Cd2—O11—C31—C32	173.9 (3)
O10—Cd2—N4—C13	146.3 (2)	Cd2—O12—C31—O11	6.5 (4)
C31—Cd2—N4—C13	−67.1 (2)	Cd2—O12—C31—C32	−173.1 (3)
O12—Cd2—N4—C24	90.4 (2)	O12—Cd2—C31—O11	−174.0 (4)
O9—Cd2—N4—C24	−76.0 (2)	O9—Cd2—C31—O11	−37.8 (3)
O8—Cd2—N4—C24	−147.8 (2)	O8—Cd2—C31—O11	42.9 (2)
N5—Cd2—N4—C24	3.39 (18)	N4—Cd2—C31—O11	126.9 (2)
O11—Cd2—N4—C24	141.21 (18)	N5—Cd2—C31—O11	−155.60 (19)
O10—Cd2—N4—C24	−32.9 (3)	O10—Cd2—C31—O11	−73.5 (2)
C31—Cd2—N4—C24	113.7 (2)	O9—Cd2—C31—O12	136.2 (2)
O12—Cd2—N5—C22	78.2 (3)	O8—Cd2—C31—O12	−143.1 (2)
O9—Cd2—N5—C22	−76.1 (2)	N4—Cd2—C31—O12	−59.1 (2)
O8—Cd2—N5—C22	−137.2 (2)	N5—Cd2—C31—O12	18.4 (3)
N4—Cd2—N5—C22	178.9 (3)	O11—Cd2—C31—O12	174.0 (4)
O11—Cd2—N5—C22	53.2 (3)	O10—Cd2—C31—O12	100.4 (2)
